# Some Practical Points in Abdominal Surgery

**Published:** 1891-07

**Authors:** Jno. H. M’Intire

**Affiliations:** 614 Olive Street; St. Louis, Mo.


### SOME PRACTICAL POINTS IN ABDOMINAL SURGERY.

BY JNO. H. M'INTIRE, A. M., M. D., ST. LOUIS, MO.

Read Before the State Medical Association of Missouri, Excelsior Springs, Mo., May 21, 1891.

"Suppose I give a hint to you,
Suppose you give a point to me;
Then I shall give a hint to you,
And you will give a point to me"—

in the discussion which I hope will follow the reading of this paper.

In my opinion, any "point" or suggestion which diminishes the risk to life after laparotomy, is an important one.

The first point to which I call your attention, is that of anasthetics, the safest and best of which is bi-chloride of methlene used in Junker's inhaler. I have used it in laparotomy work for the past ten years without a single untoward symptom, and with the greatest satisfaction, and upon many occasions have put it to as severe a test as it is possible to put an anaesthetic.

By its use, anasthesia cannot only be promptly induced, but safely maintained for any desirable length of time, and it is rarely followed by nausea and vomiting.

By the use of the inhaler of Junker, overdosing is next to impossible, in reality the patient takes inspired air, charged with the vapor of bi-chloride of methylene, and it is surprising what a small quantity is required in doing a prolonged operation.

Short incisions constitute another point of excellence, and should never be extended beyond the point of necessity in removing a growth of given size without bruising the tissues. In removing ovaries or fallopian tubes, or both, it is rarely that the ventral incision need be over two inches in extent.

In dealing with adhesions, perseverance by well directed effort will always succeed; remembering however, that violence is always harmful, and the necessary force should be that of gentle momentum.

Intestinal adhesions, should be separated as far from the gut as possible, for by so doing the danger of hemorrhage is much lessened; they should be carelly examined afterward, as the placing of a Lembert suture in the proper place at the opportune moment, will prevent the mortification of a future fecal fistula.

In the management of the pedicle, I always use Japanese cable silk, transfixing and tying the ordinary surgical knot, when dealing with large tumors; for removal of the appendages, I am partial to the Staffordshire Knot of Tait.

DRAINAGE.

"When in doubt" I always drain and prefer the Keith tube to all others, and am a thorough believer in flushing the abdomen with a large quantity of hot distilled water; it is marvelous sometimes to see how many blood clots can thus be washed out, even after careful sponging, besides it is one of the best methods of relieving shock.

Closure of ventral wound can best be done with silk worm gut; it is the ideal suture, as it is round, smooth and very strong, and can be rendered perfectly aseptic. As it is rather stiff, it should be steeped for a few hours before using, in a solution of some kind, so that it can be tied tightly. It should be threaded at each end upon straight or slightly curved veterinary needles. The needle being held in grasp of the Spencer-Wells needle holder, should be passed from within outward, always including the peritoneum. Sutures should be placed five or six to the inch. The frequent cause of ventral hernia following abdominal section, is an insufficient number of sutures.

AFTER MANAGEMENT.

For the first twenty-fours, nothing should be taken into the stomach, except a little hot water; bits of ice chewed or swallowed do not relieve thirst. The second day a little barley water may be allowed, and on the third day she can be promoted to a chicken wing, when afterwards, if every thing goes well, almost any light diet may be allowed.

When pain is present, I use but little morphia, on account of its tendency to arrest secretions, and thereby prevent the elimination of morbid material, but in its stead, for more than a year past, have used antikamnia, with happy effect. It soothes and tranquilizes, and lessens the tendency to rise of temperature.

Stitch hole sinuses can best be obviated by the early removal of the sutures. It is rarely that I allow sutures to remain in the ventral wound longer than the eighth day, and I often remove them as early as the sixth.

He who essays to do abdominal and pelvic operations, should by previous observation and training, be so fitted for his work that when he comes into "action" he will be "ready for anything, and surprised at nothing."

The best place in which to attain the highest grade of success, is not in large general hospitals, neither is it in "the cottage by the wayside," but in a small especially prepared establishment, under the absolute control of experienced mnnagement.

*614 Olive Street.*

---

ŒODEMA OF THE GLOTTIS FOLLOWING INFLUENZA.—Before the Imperial Society of Medicine of Constantinople, Bavachi (*Gazette Med. d'Orient*, April 15, 1891) reported three cases of influenza in which œdema of the glottis developed. One was in a man of fifty, the second in a man of thirty, and the third in a girl of three. The first died, the general condition not being favorable for tracheotomy; the other two recovered without operative interference.—*Ex.*

